# Supplementary material for: Multi‐dataset identification of innovative feature genes and molecular mechanisms in keratoconus
Source: J Cell Mol Med. 2024 Sep 19;28(18):e70079. doi: 10.1111/jcmm.70079 (PMC11412914; doi:10.1111/jcmm.70079)
Supplement: Supplementary file 2 — Material S2. [file JCMM-28-e70079-s002.zip › 7.GSEA analysis/3.DBNDD1/KEGG_FC_GAMMA_R_MEDIATED_PHAGOCYTOSIS.html]

Details for gene set KEGG\_FC\_GAMMA\_R\_MEDIATED\_PHAGOCYTOSIS[GSEA]

|  || Dataset | DBNDD1.group.cls#h\_versus\_l |
| Phenotype | group.cls#h\_versus\_l |
| Upregulated in class | l |
| GeneSet | KEGG\_FC\_GAMMA\_R\_MEDIATED\_PHAGOCYTOSIS |
| Enrichment Score (ES) | -0.38029453 |
| Normalized Enrichment Score (NES) | -1.5073404 |
| Nominal p-value | 0.055555556 |
| FDR q-value | 0.70429474 |
| FWER p-Value | 0.787 |
Table: GSEA Results Summary

  

Fig 1: Enrichment plot: KEGG\_FC\_GAMMA\_R\_MEDIATED\_PHAGOCYTOSIS      
 Profile of the Running ES Score & Positions of GeneSet Members on the Rank Ordered List

  

| SYMBOL | TITLE | RANK IN GENE LIST | RANK METRIC SCORE | RUNNING ES | CORE ENRICHMENT || 1 | PIK3R5 | na | 35 | 0.708 | 0.0692 | No |
| 2 | PLA2G4D | na | 439 | 0.469 | 0.1040 | No |
| 3 | FCGR1A | na | 3835 | 0.219 | 0.0277 | No |
| 4 | HCK | na | 4340 | 0.202 | 0.0332 | No |
| 5 | INPP5D | na | 4602 | 0.193 | 0.0448 | No |
| 6 | PRKCG | na | 6020 | 0.150 | 0.0188 | No |
| 7 | ASAP3 | na | 6521 | 0.136 | 0.0178 | No |
| 8 | PIK3R1 | na | 6912 | 0.126 | 0.0191 | No |
| 9 | ARPC4 | na | 7032 | 0.124 | 0.0279 | No |
| 10 | FCGR2A | na | 8721 | 0.090 | -0.0119 | No |
| 11 | FCGR2B | na | 8722 | 0.090 | -0.0029 | No |
| 12 | AKT2 | na | 9388 | 0.080 | -0.0142 | No |
| 13 | PIP4K2B | na | 9747 | 0.075 | -0.0171 | No |
| 14 | MAPK3 | na | 9865 | 0.073 | -0.0132 | No |
| 15 | VAV2 | na | 10635 | 0.063 | -0.0292 | No |
| 16 | GSN | na | 11017 | 0.058 | -0.0344 | No |
| 17 | LIMK1 | na | 11402 | 0.053 | -0.0402 | No |
| 18 | PIK3R2 | na | 11699 | 0.050 | -0.0438 | No |
| 19 | PLCG1 | na | 11955 | 0.048 | -0.0465 | No |
| 20 | SPHK1 | na | 13199 | 0.037 | -0.0787 | No |
| 21 | PLA2G6 | na | 14048 | 0.030 | -0.1003 | No |
| 22 | PIKFYVE | na | 14064 | 0.030 | -0.0978 | No |
| 23 | ARPC3 | na | 14123 | 0.029 | -0.0966 | No |
| 24 | PIK3R3 | na | 14144 | 0.029 | -0.0943 | No |
| 25 | RPS6KB2 | na | 14214 | 0.028 | -0.0935 | No |
| 26 | RPS6KB1 | na | 14385 | 0.027 | -0.0957 | No |
| 27 | RAC1 | na | 14759 | 0.025 | -0.1040 | No |
| 28 | PLD1 | na | 15421 | 0.020 | -0.1211 | No |
| 29 | ARPC1A | na | 15571 | 0.019 | -0.1236 | No |
| 30 | PLA2G4A | na | 15676 | 0.018 | -0.1248 | No |
| 31 | MYO10 | na | 15934 | 0.016 | -0.1306 | No |
| 32 | WASF2 | na | 16020 | 0.016 | -0.1315 | No |
| 33 | PIP5K1C | na | 16052 | 0.015 | -0.1309 | No |
| 34 | VASP | na | 16674 | 0.011 | -0.1477 | No |
| 35 | VAV3 | na | 16809 | 0.011 | -0.1505 | No |
| 36 | PTPRC | na | 16868 | 0.010 | -0.1511 | No |
| 37 | PIK3CA | na | 17073 | 0.009 | -0.1561 | No |
| 38 | ARPC2 | na | 17961 | 0.003 | -0.1814 | No |
| 39 | DNM2 | na | 18068 | 0.002 | -0.1843 | No |
| 40 | WAS | na | 18085 | 0.002 | -0.1846 | No |
| 41 | PIK3CB | na | 18338 | 0.000 | -0.1918 | No |
| 42 | ARPC5L | na | 18383 | 0.000 | -0.1931 | No |
| 43 | RAF1 | na | 19005 | -0.005 | -0.2105 | No |
| 44 | WASF1 | na | 19038 | -0.005 | -0.2110 | No |
| 45 | AKT1 | na | 19066 | -0.005 | -0.2113 | No |
| 46 | PLCG2 | na | 19340 | -0.007 | -0.2184 | No |
| 47 | SYK | na | 19415 | -0.008 | -0.2198 | No |
| 48 | PLA2G4E | na | 19468 | -0.008 | -0.2205 | No |
| 49 | ARF6 | na | 19486 | -0.008 | -0.2201 | No |
| 50 | CFL1 | na | 19621 | -0.010 | -0.2230 | No |
| 51 | PLA2G4F | na | 19688 | -0.010 | -0.2239 | No |
| 52 | LIMK2 | na | 20036 | -0.013 | -0.2326 | No |
| 53 | PLD2 | na | 20094 | -0.014 | -0.2329 | No |
| 54 | WASL | na | 20105 | -0.014 | -0.2318 | No |
| 55 | ARPC5 | na | 20113 | -0.014 | -0.2306 | No |
| 56 | ARPC1B | na | 20430 | -0.017 | -0.2381 | No |
| 57 | PRKCD | na | 20754 | -0.020 | -0.2455 | No |
| 58 | RAC2 | na | 20865 | -0.021 | -0.2466 | No |
| 59 | DNM1L | na | 21040 | -0.022 | -0.2494 | No |
| 60 | PRKCA | na | 21412 | -0.026 | -0.2575 | No |
| 61 | CDC42 | na | 22626 | -0.039 | -0.2887 | No |
| 62 | MAPK1 | na | 22705 | -0.040 | -0.2870 | No |
| 63 | DNM1 | na | 23055 | -0.044 | -0.2927 | No |
| 64 | CRK | na | 23147 | -0.045 | -0.2909 | No |
| 65 | WASF3 | na | 24176 | -0.059 | -0.3148 | No |
| 66 | PIK3CD | na | 24971 | -0.070 | -0.3307 | No |
| 67 | MARCKS | na | 26276 | -0.093 | -0.3591 | No |
| 68 | CRKL | na | 27010 | -0.108 | -0.3696 | Yes |
| 69 | NCF1 | na | 27044 | -0.109 | -0.3597 | Yes |
| 70 | FCGR3A | na | 27389 | -0.116 | -0.3581 | Yes |
| 71 | PIK3CG | na | 27455 | -0.118 | -0.3483 | Yes |
| 72 | ASAP2 | na | 27677 | -0.123 | -0.3425 | Yes |
| 73 | PAK1 | na | 28053 | -0.132 | -0.3402 | Yes |
| 74 | DNM3 | na | 28124 | -0.134 | -0.3289 | Yes |
| 75 | LYN | na | 28223 | -0.136 | -0.3183 | Yes |
| 76 | SPHK2 | na | 28315 | -0.138 | -0.3072 | Yes |
| 77 | SCIN | na | 28853 | -0.153 | -0.3076 | Yes |
| 78 | VAV1 | na | 28859 | -0.153 | -0.2926 | Yes |
| 79 | MAP2K1 | na | 29246 | -0.164 | -0.2874 | Yes |
| 80 | FCGR2C | na | 29669 | -0.178 | -0.2820 | Yes |
| 81 | LAT | na | 29791 | -0.182 | -0.2674 | Yes |
| 82 | AMPH | na | 30518 | -0.204 | -0.2681 | Yes |
| 83 | CFL2 | na | 31245 | -0.230 | -0.2663 | Yes |
| 84 | PIP5K1A | na | 32090 | -0.268 | -0.2640 | Yes |
| 85 | GAB2 | na | 32145 | -0.272 | -0.2386 | Yes |
| 86 | PRKCE | na | 32256 | -0.280 | -0.2140 | Yes |
| 87 | MARCKSL1 | na | 32319 | -0.283 | -0.1877 | Yes |
| 88 | AKT3 | na | 33656 | -0.385 | -0.1882 | Yes |
| 89 | ASAP1 | na | 34095 | -0.442 | -0.1569 | Yes |
| 90 | PRKCB | na | 34153 | -0.452 | -0.1138 | Yes |
| 91 | DOCK2 | na | 34391 | -0.511 | -0.0699 | Yes |
| 92 | PIP5K1B | na | 34702 | -0.803 | 0.0008 | Yes |
Table: GSEA details [plain text format]

  

Fig 2: KEGG\_FC\_GAMMA\_R\_MEDIATED\_PHAGOCYTOSIS      
 Blue-Pink O' Gram in the Space of the Analyzed GeneSet

  

Fig 3: KEGG\_FC\_GAMMA\_R\_MEDIATED\_PHAGOCYTOSIS: Random ES distribution      
 Gene set null distribution of ES for **KEGG\_FC\_GAMMA\_R\_MEDIATED\_PHAGOCYTOSIS**
